# A Comparative Analysis of Eye Movement Accuracy for Locating Items Held in Visual Short‐Term Memory Among Young Healthy Adults, Older Adults With Normal Cognition, and Older Adults Indicative of Mild Cognitive Impairment

**DOI:** 10.1002/brb3.71021

**Published:** 2025-11-05

**Authors:** Raju Sapkota, Monika McAtarsney‐Kovacs, Ian van der Linde, Shahina Pardhan

**Affiliations:** ^1^ Vision & Eye Research Institute, School of Medicine Anglia Ruskin University Cambridge UK; ^2^ School of Computing and Information Science Anglia Ruskin University Cambridge UK; ^3^ Cognition and Neuroscience Group, ARU Centre for Mind and Behaviour Anglia Ruskin University Cambridge UK

**Keywords:** eye movements, mild cognitive impairment, object‐location binding, transsaccadic memory, visual short‐term memory

## Abstract

**Background:**

We compared the accuracy of eye movements in locating an item stored in visual short‐term memory between young healthy adults, normally aging older adults, and older adults with mild cognitive impairment as indicated by the Montreal Cognitive Assessment or Addenbrooke's Cognitive Examination‐III test.

**Methods:**

Thirty‐three young healthy adults, 38 normally aging older adults, and 17 older adults indicative of MCI completed two experiments requiring object‐location binding. In Experiment 1, participants viewed 2–4 memory items displayed sequentially at random screen locations. Following a 900 ms interval, eye movements were recorded while participants moved their eyes to the location of the memory item corresponding to a displayed cue. In Experiment 2 (control), participants indicated whether or not the test item was shown at its original location using a yes/no response.

**Results:**

MCI‐indicative participants exhibited greater saccadic error (spatial deviation of saccadic endpoint from the remembered target location) than normally aging older (*p* = 0.002) and young (*p* < 0.001) participants at low memory load only. At higher memory load, the saccadic error distance was greater for all groups (*p* < 0.001). Moreover, in Experiment 2, MCI‐indicative participants exhibited significantly poorer memory performance than normally aging older adults, but only at lower memory load (*p* = 0.02).

**Conclusion:**

Saccadic accuracy declined with memory load for all groups. The MCI‐indicative group showed lower saccadic accuracy versus normally aging older and young adults at low memory load. The findings offer important insights into our understanding of saccadic eye movement as a potential behavioral marker for MCI.

## Graphical abstract

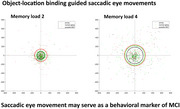

## 1 Introduction

Visual short‐term memory (VSTM) is a transient memory system that lasts for up to a few seconds (Phillips 1974) and supports everyday tasks such as change detection, visual search, object tracking, and scene navigation (Hollingworth et al. 2008). When VSTM is used in guiding eye movements, it is known as “transsaccadic memory” (Hollingworth et al. 2008; Irwin 1991; Irwin and Andrews 1996), which helps maintain perceptual continuity by integrating visual information across saccades, thereby compensating for the transient disruption in retinal input caused by the saccades (Treisman 1988).

Central to the VSTM is the concept of *object file*, which is a spatially indexed mental representation encoding individual items from the visual field in VSTM (Kahneman et al. 1992). Object files support object‐location binding, a cognitive process that enables our visual system to retain information about ‘what’ object is located ‘where’ in the space (Hollingworth and Rasmussen 2010; Triesman 1988). Object files are refreshed, updated, or overwritten depending upon whether an item is re‐examined at the same location, a new location, or replaced by a different item (Kahneman et al. 1992).

The slot model account of VSTM suggests that object files are represented in fixed‐capacity slots (up to 3–4) (Cowan 2005; Luck and Vogel 1997; Vogel et al. 2001), depending upon the complexity and familiarity of items (Alvarez and Cavanagh 2004; Olsson and Poom 2005; Sapkota et al. 2011). On the other hand, the resource model account of VSTM suggests that object‐file representation is not constrained by a fixed item limit but instead reflects a shared pool of cognitive resources that can be flexibly distributed across multiple items, with precision decreasing as more or complex items are stored (Bays et al. 2009; Wilken and Ma 2004).

Besides the object familiarity and complexity, age is also known to influence VSTM, with performance declining progressively with advancing age (Mitchell et al. 2000; Sapkota et al. 2015; Sapkota et al. 2023). This decline is thought to be associated with age‐related structural deteriorations mainly in hippocampal brain areas, which are known to play a crucial role in mediating object‐location binding (Backman et al. 1999; Wheeler and Triesman 2012). Evidence suggests that the hippocampal structural changes are greater in people who exhibit mild cognitive impairment, MCI (Du et al. 2001; Hampel et al. 2008), a transitional stage between normal cognitive aging and dementia (Petersen et al. 1999). Furthermore, the hippocampal areas are interconnected with the oculomotor system through extensive structural and functional networks (Ryan and Shen 2020).

Saccadic eye movements are found to be generally slower and less precise with advancing age (Moschner and Baloh 1994; Dowiasch et al. 2015). A study by Yang and colleagues (2013) examined horizontal and vertical saccades in healthy elderly individuals, MCI, and Alzheimer's disease (AD) patients using tasks in which the fixation target was either extinguished or persisted after target onset. AD patients were found to show significantly longer and more variable saccade latencies, especially compared to healthy controls and MCI participants. Pereira et al. (2020) analyzed eye movement behavior in adults with MCI, AD, and control normal subjects while they performed an anti‐saccade task, in which they were required to inhibit a prosaccade towards the target stimulus and initiate a voluntary saccade in the opposite direction, reporting that MCI and AD patients showed significantly increased response times.

A few studies have examined eye movements during a VSTM binding task in individuals with normal cognitive aging, MCI, and early dementia. Shih and colleagues (2012) conducted a series of experiments in which healthy young and older adults were presented with memory items at different spatial locations on a computer screen and were later tested for their ability to recall those locations. Eye movements were measured while participants examined the memory items. Older adults exhibited inferior performance in the location recall task relative to younger adults, which correlated with a reduced number of fixation counts on the memory target, potentially resulting in being less effectively encoded in VSTM. Fernández et al. (2018) examined eye movements in people diagnosed with a mild form of Alzheimer's disease by displaying consecutive arrays of two bicolored objects and requiring participants to perform a VSTM task for individual colors or their bound form. They reported a significant reduction in VSTM performance with trials that required memory for bound colors, which was associated with shorter stimulus fixation durations.

Previous studies on VSTM binding have primarily examined eye movements during the memory encoding stage (i.e., while participants examined items in the memory display), with comparatively little attention given to saccadic eye movements during memory retrieval. Retrieval‐related saccades may reflect how well spatial information stored in VSTM is accessed and translated into eye movements for shifting gaze from one target to another. Unlike encoding, which is largely influenced by the visual (external) item (Uncapher et al. 2011; Wynn et al. 2019), the retrieval of the position associated with that item depends upon the internal reconstruction of object‐location binding (Markov and Utochkin 2022). Investigating saccadic behavior during the retrieval phase may offer critical insights into the underlying cognitive mechanisms guiding saccades, an area of research that remains unexplored. From a practical standpoint, saccades during the memory retrieval phase may serve as sensitive behavioral markers of cognitive decline, indicated by inaccurate or delayed saccadic responses, even when stimulus encoding appears intact. Exploring this further is likely to provide significant insights into the clinical utility of saccadic eye movement as a potential behavioral marker of early cognitive decline associated with normal aging and dementia. In most previous studies that examined eye movements during a VSTM binding task, two or more stimuli were presented simultaneously. It is worth pointing out that when multiple items are viewed simultaneously, VSTM is likely to be influenced by interference from peripheral items, which individuals with MCI are less likely to be able to inhibit (Aurtenetxe et al. 2016). Therefore, the effect of MCI on eye movement accuracy in shifting gaze towards a memorized location, without incurring interference from simultaneously displayed peripheral items, remains unclear, an issue that was examined in the current study. A paradigm that required participants (young healthy adults, normally aging older adults, and older adults indicated to have MCI; see “Methods” section) to retrieve object‐location binding from VSTM was used. Participants were asked to shift their gaze to the location where they believed the test item had been shown in the memory display, and their responses were recorded using an eye tracker. The hypothesis tested was that eye movement inaccuracy appears earlier than VSTM decline in the MCI group, and this is associated with a reduced ability to retrieve object‐location binding from VSTM. Items were presented sequentially, one at a time, to avoid interference arising from simultaneously viewed peripheral items. A control experiment was run to examine how VSTM performance (% correct) for retrieving object‐location binding differed between the participant groups. The findings will shed light on whether reduced saccadic accuracy among adults with MCI emerges earlier than VSTM decline for object‐location binding, pointing to saccadic inaccuracy as a potentially more sensitive early behavioral marker of MCI.

## 2 Experiment 1

### 2.1 Methods

#### 2.1.1 Participants

Thirty‐seven young healthy adults (mean age = 24.7 ± 2.9 years), 63 older adults (mean age = 70.2 ± 6.4 years) meeting threshold criteria for normal cognition in a standard Montreal Cognitive Assessment (≥ 26) (Nasreddine et al. 2005) and Addenbrooke's Cognitive Examination‐III (ACE‐III) tests (> 88) (Hsieh et al. 2013), and 24 older adults (mean age = 72.4 ± 6.5 years) satisfying cognitive score criteria for indicating MCI (*hereafter referred to as MCI‐indicative*) on either MoCA (range: 18–25) or ACE‐III (range: 75–88) tests were initially invited to take part in VSTM experiments. After accounting for those with incomplete trials or not having ‘good’ calibration with the eye tracker, the final sample of participants was as follows: 33 young adults (mean age = 24.7 ± 2.8 years), 38 older adults (mean age = 68.0 ± 6.3 years), and 17 MCI‐indicative adults (mean age = 71.7 ± 6.3 years). The excluded sample in each group did not differ significantly in terms of age, gender, education level, and MoCA or ACE‐III scores compared to the included sample.

The size of the effect of MCI on object‐location binding‐guided saccadic behavior is not precisely known, as no prior studies have directly investigated this topic. Hence, the exact sample size calculation was not possible. However, our sample size is greater than studies by (i) Sapkota et al. (2017), which examined the impact of MCI on object‐location binding. 10 MCI participants were employed, and (ii) Fernández et al. (2018), which examined the impact of mild dementia on fixational eye movement for encoding color‐color binding, with 13 participants.

Participants were invited from the Join Dementia Research platform, the University of the Third Age (U3A), and by circulating posters and flyers at the Cambridge campus of Anglia Ruskin University and local communities, including a retirement home.

All participants had normal or corrected‐to‐normal vision (≥ 6/6). Individuals with the following self‐reported conditions were excluded: uncorrected visual and hearing impairment (participants using glasses and hearing aids were included), color vision defect, known history of neurological or psychological disorders (except MCI), brain injury, strabismus, epilepsy, dyslexia, stroke, degenerative diseases such as Parkinson's, depression, anxiety, and chronic alcohol and drug use. All participants were able to speak English fluently (native and non‐native). Table 1 provides a breakdown of participants’ demographic characteristics.

**TABLE 1:** Demographic characteristics of participant groups.

Demographic variable		Young adults	Cognitively normal older adults	MCI‐indicative adults
Age in years (mean ± SD, median)		24.7 ± 2.8, 24.0	68.0 ± 6.3, 69.0	71.7 ± 6.3, 71.0
Years of education (mean ± SD, median)		18.1 ± 2.3, 17.0	17.3±3.4, 16.5	16.4 ± 2.9, 17.0
Gender:		%	%	%
	Male	36.4%	52.6%	52.9%
	Female	63.6%	47.4%	47.1%

All participants were treated in accordance with applicable ethical guidelines that followed the tenets of the Helsinki Declaration. The study protocol was approved by the ethical approval from the School Research Ethics Panel (SREP approval number CIS201221), Faculty of Science and Engineering, Anglia Ruskin University. Participants were reimbursed for their travel expenses.

#### 2.1.2 Stimuli and Apparatus

**FIGURE 1:**
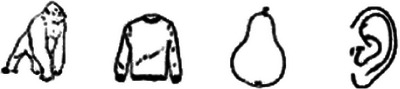
Type of stimuli used.

The stimuli comprised 180 line drawings of real‐world objects (Snodgrass and Vanderwart 1980), each subtending 2.5° of the visual angle at a 57 cm distance (suprathreshold stimuli). Example stimuli are shown in Figure 1. Nameable stimuli were used for ecological validity. Stimuli were displayed using a laptop computer (Dell Precision, M4500) connected to an external display (Sony, GDM‐F520) with a screen resolution set at 1440 × 900 pixels and a refresh rate of 60 Hz. The central 750 × 750‐pixel area was used to display stimuli. The experiments were implemented in SR Research Experiment Builder software version 2.3.38. A high‐accuracy video‐based eye tracker (SR Research EyeLink 1000, Desktop Mount) was used to record eye movements during object‐location retrieval. Participants were sitting in a dark room with their head positioned on a chin and forehead rest to minimize head movement.

Before the experiment, participants completed standard paper‐based cognitive tests, namely ACE‐III and MoCA. Those satisfying the test score criteria for MCI in ACE‐III (≤ 88) (Hsieh et al. 2013) or MoCA (≤ 25) (Nasreddine et al. 2005) were classified as MCI‐indicative. While both tests (ACE‐III and MoCA) are widely used screening instruments for dementia, which of the two tests is superior for screening MCI is debated and beyond the scope of this study (Li et al. 2019; Senda et al. 2020; Valles‐Salgado et al. 2024; Abd Razak et al. 2019; Tsoi et al. 2015). Therefore, we used both test scores to group participants as cognitively normal young adults, cognitively normal older adults, or MCI‐indicative older adults.

#### 2.1.3 Eye Tracker Calibration and Validation

A standard nine‐point calibration and validation were conducted for each participant to align eye and screen coordinates, in which the visual cues (dots) appeared automatically on the screen. The calibration sequence was labeled as good, where the average error was less than 1° (Vadillo et al. 2015). Only data from participants with good calibration and validation were included in the analysis (average error: < 1.0°, maximum error: < 1.5°, Kaduk et al. 2024).

#### 2.1.4 Experimental Procedure

**FIGURE 2:**
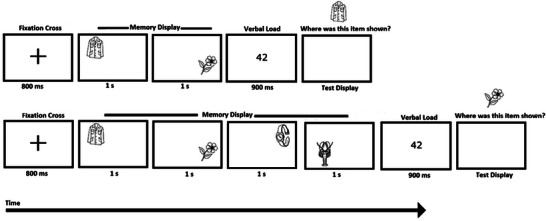
Schematic diagram of an example trial sequence in Experiment 1 with a memory load of two items (above) and four items (below).

Experimental procedures were based on our previous studies (Sapkota et al. 2017, 2020). Figure 2 provides a schematic representation of the experimental procedure used.

Each trial began with a fixation cross displayed at the center of the screen for 800 ms. Next, either two or four stimuli were presented sequentially, each displayed for 1000 ms at any of the 25 random locations (computer‐generated) within a 750 × 750‐pixel grid centered on the screen. Memory items appeared at least one stimulus size (150 × 150 pixels) apart from each other. Participants were required to remember both memory items and their locations. This was followed by the presentation of a random two‐digit number for 900 ms that participants had to read out loud to discourage verbal retention of the stimuli and recruitment of long‐term memory support (articulatory suppression) (Baddeley and Hitch 1974; Todd and Marois 2004). Next, a test display was presented, in which participants were shown one of the memory items at the top of the display screen (i.e., not at its original location) and were asked, ‘Where was this object shown?’. Participants were asked to respond to the question by executing a saccade to the location at which they thought they had seen the test item in the preceding memory display (saccadic endpoint). The accuracy of the saccadic endpoint was recorded using an eye tracker (the left eye was tracked for all participants). Once participants were satisfied with their selection, they were asked to click the computer mouse (i.e., a non‐speeded task was used), initiating the next trial. Distances (*d*) between the actual location of the test item in the memory display and the location of the saccadic endpoint to retrieve its memorized location were calculated as 2D Euclidean distance (Equation 1).

(1) $$d=\sqrt{{\left(X_{2}-X_{1}\right)}^{2}+{\left(Y_{2}-Y_{1}\right)}^{2}}$$

where X_1_ and Y_1_ are the coordinates of the original location, and X_2_ and Y_2_ are the coordinates of the saccadic endpoint, which may include corrective saccades. For convenience, hereafter, this distance (*d*) is referred to as the *saccadic error distance*. The lower the value of *d*, the greater the accuracy of the saccade ("0" being a saccade landing exactly on the center of the location at which the test item was shown in the preceding memory display).

Participants were told through the experimenter and on‐screen verbal instructions that in any given trial, there would be either two or four (randomly interleaved) memory items selected randomly by the computer, presented at random spatial locations on the display screen. Object‐location memory would be tested by displaying one of the randomly selected memory items (a retrieval cue), shown at a fixed location at the display screen top (Experiment 1), and asking participants to recall the location at which the test item had been previously shown by shifting their gaze to that location. Participants would not have prior knowledge of which item or memory load (two or four items) was going to be tested, hence requiring them to remember all stimuli without focusing on one specific memory load or item. Once participants were satisfied with the clarity of the instructions given to them, they did four practice trials (or more if necessary), followed by experimental trials.

There were 16 trials per participant, divided equally between the two memory loads, i.e., memory load 2 (ML2) and memory load 4 (ML4), in line with the published protocol (Sapkota et al. 2017; Sapkota et al. 2020). Although we initially aimed to collect data from 32 trials per participant, the increase in overall test time proved challenging for some elderly participants. Consequently, we adhered to the original experimental protocol of 16 trials, equally divided across memory loads 2 and 4. Temporal positions from which the test item was selected were tested equally often, i.e., for ML2, each of the two temporal positions was tested four times; for ML4, each of the four temporal positions was tested twice. Participants could take as many breaks as required. Participants had four practice trials to familiarize themselves with the procedure at the beginning of the test, which were not included in the analysis. It took approximately 45 min per participant to complete the paper‐based cognitive tests (ACE‐III or MoCA) and the VSTM binding tests, including the calibration and validation of the eye‐tracker and a quick vision assessment. Where data were not normally distributed (*p* < 0.05, Shapiro–Wilk test), nonparametric tests were used, such as Kruskal–Wallis, Mann–Whitney *U*, and Wilcoxon tests. Bonferroni correction was applied for multiple comparisons to control the family‐wise error rate. Age was included as a covariate in analyses comparing the young group to older groups. Within each participant group, saccadic error distances were normalized using the following formula.

(2) $$\begin{array}{ccc} Normalized\;error & & =(Observed\;saccadic\;error\;distance\;\\ & & ÷\;Maximum\;possible\;error)\times 100 \end{array}$$

where the maximum possible error corresponds to the greatest 2D Euclidean distance between any two memory items.

### 2.2 Results

Table 1 provides a summary of demographic data such as age, gender, and number of years of education. Participant groups did not differ significantly in the number of years of education, *H (*2, *n* = 88) = 3.2, *p* = 0.21, *η^2^p* = 0.01. A non‐significant relationship between participant groups and gender was found, *χ^2^* (2, *n* = 88) = 2.2, *p* = 0.36, *η^2^p* = 0.16. Also, there was no significant difference in age between those indicative of MCI and cognitively normal older participants (*z* = 1.7, *p* = 0.08, *η^2^p* = 0.05).

#### 2.2.1 Eye Movement Data

**FIGURE 3:**
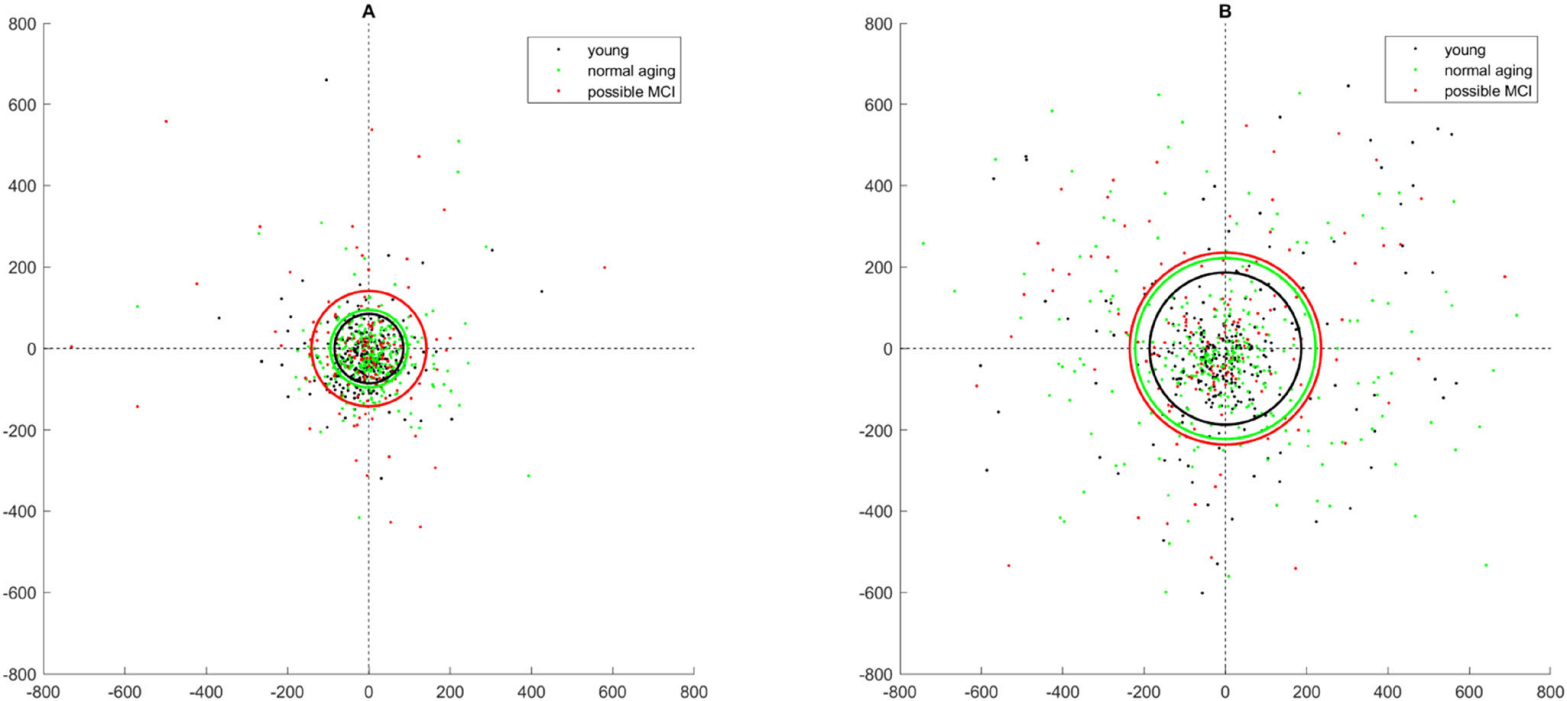
Spatial distribution of saccadic endpoint for young (black), normal aging (green), and MCI‐indicative adults (red) for ML2 (A) and ML4 (B). (0, 0) represents the original location of the target item in the memory display. The circles show the mean Euclidean distance from (0, 0).

Data confirmed good calibration accuracy (mean error = 0.44°, SD = 0.15, maximum error = 0.94°). Figure 3 shows the spatial distribution of the saccadic endpoint from the location of a memory target following a saccadic eye movement.

**FIGURE 4:**
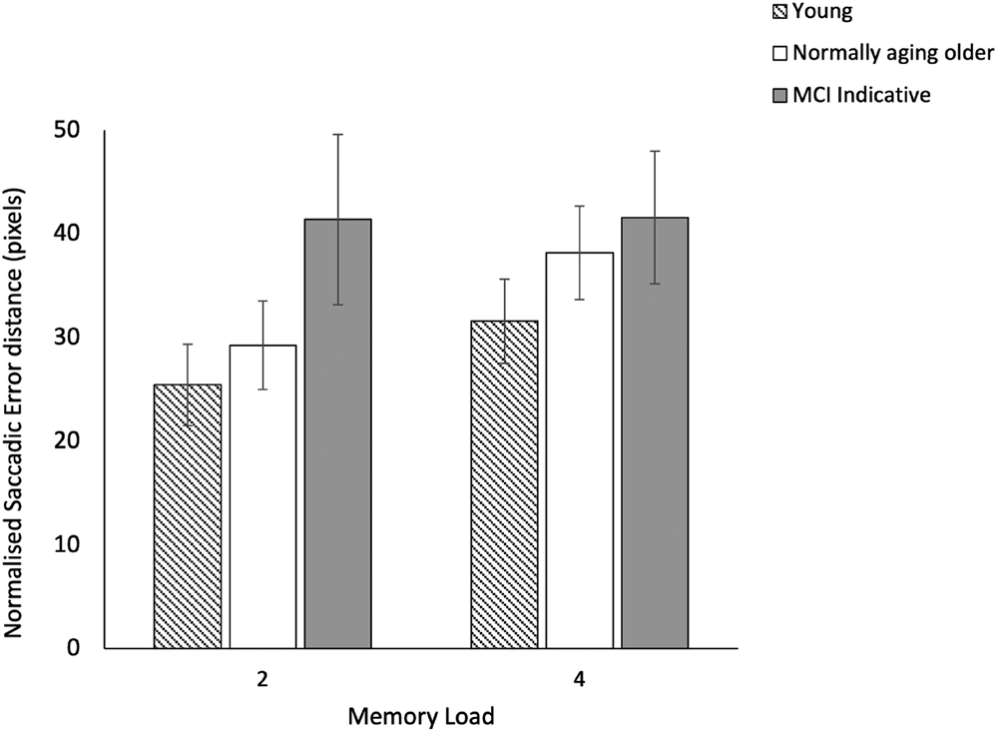
Mean normalized saccadic error distances for young, normally aging, and MCI‐indicative participants in memory load 2 and 4. Error bars represent ± 1.96 SE.

Figure 4 provides means of the normalized saccadic error distance, i.e., the distance (in pixels) between the original position at which the test item was shown in the memory display (*original location*) and the location to which participants executed a saccade to indicate where in the memory display the test item was shown for healthy young adults, normally aging older adults, and older MCI‐indicative adults.

Table 2 provides a summary of statistical test results on normalized saccadic error distances. A significant effect of participant group on saccadic error distances was found [ML2: *H* (2) = 12.4, *p* = 0.002, *η^2^p* = 0.12; ML4: *H* (2) = 7.6, *p* = 0.03, *η^2^p =* 0.07]. Post‐hoc comparisons revealed significantly greater saccadic error distances for participants indicative of MCI compared to the normally aging older participants [*U* = 180, *Z* = 2.6, *p* = 0.009, *η^2^p =* 0.12] and the young participants [*U* = 117, *Z* = 3.3, *p* < 0.001, *η^2^p* = 0.22], but only at ML2. On the other hand, significantly greater saccadic error distances for cognitively normal compared to young participants were found, but only at ML4 [*U* = 446, *Z* = 2.0, *p* = 0.04, *η^2^p =* 0.06]. Saccadic error distances were found to increase significantly with an increase in memory load from 2 to 4 items for young participants and cognitively normal older participants, but not MCI‐indicative participants (Table 2).

**TABLE 2:** Summary of statistical test results for Experiment 1, evaluating normalized saccadic error differences within and between participant groups.

Kruskal–Wallis test results: Between‐group effect			
Memory load	*H* (df)	*p‐v*alue	Effect size (*η^2^p)*
ML2	*H* (2) = 12.4	0.002	0.12
ML4	*H* (2) = 7.6	0.03	0.07
**Post‐hoc Mann–Whitney *U* tests (ML2)**:			
MCI‐indicative vs. Normally aging adults	*U* = 180, *Z* = 2.6	0.009	0.12
MCI‐indicative vs. Young adults	*U* = 117, *Z* = 3.3	< 0.001	0.22
Normally aging vs. Young adults	*U* = 501, *Z* = 1.5	0.15	0.03
**Post‐hoc Mann–Whitney *U* tests (ML4)**:			
MCI‐indicative vs. Normally aging adults	*U* = 268, *Z* = 1.0	0.32	0.02
MCI‐indicative vs. Young adults	*U* = 161, *Z* = 2.4	0.01	0.12
Normally aging vs. Young adults	*U* = 446, *Z* = 2.0	0.04	0.06
**Wilcoxon signed‐rank test results (ML2 vs. ML4): Within‐group effect**			
Group	z‐score	*p*‐value	Effect size (*η^2^p)*
Young adults	−2.3	0.02	0.16
Normally aging adults	−5.2	0.03	0.71
MCI‐indicative adults	−0.02	0.98	< 0.01

No significant effect of the test item's recency on differences in saccadic error distance between the participant groups was found (*p* > 0.05).

Although in Experiment 1 the task required participants to correctly remember the location of a test item in the memory display, participants did not explicitly perform a memory recognition task; hence, it is not possible to ascertain whether differences in saccadic error distance (and hence saccadic accuracy) between participant groups relate to differences in VSTM performance for object‐location binding or, specifically, whether saccadic accuracy decline emerges earlier than VSTM decline for object‐location binding in the MCI adults. To address this, Experiment 2 was conducted, in which the same participants who completed Experiment 1 performed a VSTM task for object‐location binding. Eye movements were not recorded in Experiment 2.

## 3 Experiment 2

### 3.1 Methods

**FIGURE 5:**
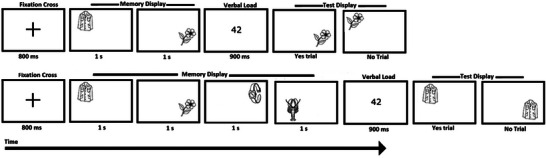
Schematic diagram of an example trial sequence in Experiment 2 with a trial sequence with a memory load of two items (above) and four items (below).

The experimental setup, stimuli display procedures, and the number of trials were similar to Experiment 1, except that eye movements were not recorded in the test display. In addition, one of the memory items was presented in the test display either at its original location (“yes” trial) or at the location of another memory item (“no” trial) (Figure 5). Participants were required to respond yes/no in response to the question ‘Was this object shown here?’ by clicking the left or right buttons of a mouse. The next trial commenced immediately after a response was submitted. Participant responses were automatically stored and grouped according to the signal detection theory categories, namely, hits, misses, false alarms, and correct rejections (Stanislaw and Todorov 1999). Memory accuracy was measured in terms of % correct responses derived by adding hits and correct rejections.

### 3.2 Results

**FIGURE 6:**
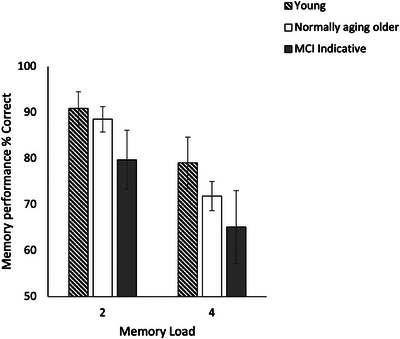
Memory performance (% (correct responses)) for the object‐location binding task in young, normally aging, and MCI‐indicative participants in ML2 and ML4. Error bars represent ± 1.96 SE.

Figure 6 provides % correct responses for different participant groups for ML2 and ML4.

Table 3 provides a summary of statistical test results on percent correct responses for Experiment 2. A significant effect of participant group on % correct responses for ML2 [*H* (2) = 8.8, *p* = 0.01, *η^2^p =* 0.08] and ML4 was found. Post‐hoc comparisons revealed significantly lower % correct responses for the MCI‐indicative group compared to the control older group, but only at ML2 [*U* = 520*, Z* = −2.3*, p* = 0.02*, η^2^p =* 0.1]. At ML4, both MCI participants and normally aging older participants performed significantly lower compared to the healthy young participants (Table 3).

**TABLE 3:** Summary of statistical test results for Experiment 2, evaluating percent correct differences within and between participant groups.

Kruskal–Wallis test results: Between‐group effect			
Memory load	*H* (df)	*p*‐value	Effect size (*η2p*)
ML2	*H* (2) = 8.8	0.01	0.08
ML4	*H* (2) = 9.4	0.009	0.09
**Post‐hoc Mann–Whitney *U* tests (ML2)**:			
MCI‐indicative vs. Normally aging adults	*U* = 520, *Z* = −2.3	0.02	0.1
MCI‐indicative vs. Young adults	*U* = 265, *Z* = −2.8	0.005	0.16
Normally Aging vs. Young adults	*U* = 1013.5, *Z* = −1.2	0.25	0.02
**Post‐hoc Mann–Whitney *U* tests (ML4)**:			
MCI‐indicative vs. Normally aging adults	*U* = 623, *Z* = –1.3	0.19	0.03
MCI‐indicative vs. Young adults	*U* = 270, *Z* = –2.6	0.009	0.13
Normally Aging vs. Young adults	*U* = 834.5, *Z* = –2.5	0.01	0.09
**Wilcoxon signed‐rank test results (ML2 vs. ML4): Within‐group effect**			
Group	z‐score	*p*‐value	Effect size (*η^2^p*)
Young adults	3.3	< 0.001	0.33
Normally aging adults	5.6	< 0.001	0.82
MCI‐indicative adults	2.6	0.005	0.39

Furthermore, trial‐level binary response data (correct/incorrect) were analyzed using Generalized Linear Mixed Models with a binomial error structure. A significant (fixed) effect of memory load, *F* (1, 1978) = 46.5, *p* < 0.001, and the participant group, *F* (2, 1978) = 11.0, *p* < 0.001, on binary (correct/incorrect) responses, was found. The interaction between the memory load and the participant group was not significant, *F* (2, 1978) = 0.11, *p* = 0.90.

The age differences in the task performance were not found to be influenced by recency effects.

Analysis of data between Experiment 1 and 2 showed that total normalized saccadic error distance correlated significantly negatively with VSTM performance for object‐location binding at ML 2 for the MCI group (*r* = −0.57, *p* = 0.02, *η^2^p* = 0.32), but not at ML4 (*r* = −0.11, *p* = 0.68, *η^2^p* = 0.01). In addition, normalized saccadic error distances were found to correlate negatively with both MoCA (*r* = −0.30, *p* = 0.005, *η^2^p* = 0.09) and ACE‐III (*r* = −0.22, *p* = 0.05, *η^2^p* = 0.01, Spearman rank test) only at ML2.

## 4 Discussion

This study investigated the accuracy of saccadic eye movements that required the retrieval of object‐location bindings from VSTM in healthy young adults, normally aging older adults, and older adults classified as MCI‐indicative. Our findings offer new insights into how aging and MCI‐indicative affect object‐location binding‐guided eye movements. In Experiment 1, significantly lower accuracy in saccadic eye movement targeting was observed in the MCI‐indicative group compared to normally aging participants, but only under ML2, suggesting that the effect of MCI is evident earlier with lower memory load. Under higher memory load, participants may rely more on spatial heuristics, such as the relative positions of objects, to guide accurate saccades. If approximate spatial configuration (formed by relative encoding of sequentially presented items) recall suffices for reasonable saccadic accuracy in all participants, this may reduce group differences at ML4. With a low memory load of just two items, spatial configuration cues may be less likely to be exploited, and individuals with MCI may struggle to retrieve VSTM representations with sufficient precision (although memory demands are minimal) to guide eye movements towards a remembered location. Future studies might consider manipulating spatial parameters, such as proportionally decreasing object sizes at ML4.

Prior study by Shih et al. (2012) showed reduced fixation counts during memory encoding in older adults; our results extend this by demonstrating that saccadic eye movements become less accurate in normally aging and MCI adults during memory retrieval. The absence of participant group differences at higher memory load (ML4), despite significant saccadic accuracy decline from ML2 to ML4 within all groups, suggests a shared upper limit in transsaccadic memory (Irwin 1991; Wilken and Ma 2004), beyond which individual differences in eye movements may have been masked.

In Experiment 1, instead of a forced‐choice test display with a suggested location of the test item, participants had to estimate its location without any spatial priming cues. This could have made the task difficult for both the older groups (MCI‐indicative and those with normal cognitive aging) for ML4 trials. Hasher and Zack (1988) conceptualized object‐location binding as a form of source memory, wherein cueing one feature of the source (e.g., its shape or color) can facilitate the retrieval of another associated feature (e.g., its location) through *feature priming*, or of the entire memory source through *source priming* (Hollingworth 2007; Kahneman et al. 1992; Sapkota et al. 2011). The absence of a spatial cue during the test display is likely to have taxed the updating of the object file through reduced *source priming*, which is reported to be compromised in normally aging older adults and MCI adults (Sapkota et al. 2017).

The slot model posits a fixed number of discrete 3–4 storage slots, each capable of holding one item with high fidelity (Cowan 2005; Luck and Vogel 1997). The memory load of four items may have exceeded the maximum number of available slots (i.e., storage capacity); consequently, only some items secured representation in the slots, which may have masked group‐level differences. The resource model, on the other hand, suggests that memory precision declines as more items are stored due to the flexible distribution of a shared pool of cognitive resources (Bays et al. 2009). The disproportionately greater saccadic error in the MCI‐indicative group at ML2 compared to other groups suggests that their object‐location memory precision may be compromised earlier, before the theoretical capacity (of 3–4 items) is reached. This supports the notion that MCI may affect not just the number of object‐location bindings stored within VSTM, but also the quality of their representations. Such an explanation is more consistent with the resource model, which allows for variability in fidelity of memory representations.

In Experiment 2, which employed a VSTM object‐location binding task without eye tracking, both normally aging and suspected MCI participants exhibited reduced performance compared to young participants, especially with higher memory load. Parra et al. (2009, 2019) found that the color‐shape binding task is sensitive to early cognitive decline, while remaining relatively unaffected by normal aging.

Notably, however, MCI‐indicative participants did not perform significantly worse than normally aging older adults at ML4, contrasting with previous findings (e.g., Sapkota et al. 2017), which is likely to be because MCI‐indicative participants in this study had a much milder form of cognitive impairment (mean ACE‐III score = 87) compared to Sapkota et al. (2017), in which clinically diagnosed MCI participants with a more severe form of cognitive impairment (mean ACE‐III score = 79.3) were studied. An additional analysis including MCI‐indicative participants with lower cognitive scores, indeed, revealed a statistically significant decline in object‐location binding performance compared to normally aging older adults (*p* < 0.05).

Our data demonstrate a significant effect of MCI on saccadic eye movements when locating a remembered item in VSTM. Our data also suggest that this may manifest earlier, with a memory load of two items.

Our findings are consistent with previous studies indicating that object‐location binding deteriorates with age (Mitchell et al. 2000; Sapkota et al. 2015), and more so in individuals with MCI (Fernández et al. 2018; Parra et al. 2010). Prior studies (Chehrehnegar et al. 2019; Opwonya et al. 2023) have used antisaccade or fixation latency measures to identify MCI from healthy aging. While earlier studies primarily focused on the analysis of eye movements during encoding, we analyzed eye movements during the memory retrieval (or test) period, an area less frequently explored. Our data suggest that saccadic accuracy during object‐location retrieval from VSTM may also offer a sensitive measure of identifying MCI from normal cognitive aging.

Our MCI‐indicative group included individuals meeting MoCA thresholds but lacking formal clinical diagnoses. This may have introduced heterogeneity, potentially diluting observable differences. Future work will confirm the data with diagnosed individuals with MCI. The sample size for the MCI‐indicative group was relatively small. The practical challenges of recruitment of MCI are well documented in the literature (Brambilla et al. 2021). We did not record the eye movements during the encoding phase. However, because only a single stimulus was presented at any given time, this would enable stimulus encoding without incurring interference from the distractor items (Zelinsky and Loschky 2005). Nonetheless, we acknowledge that incorporating eye‐tracking during the encoding phase could have yielded more nuanced insights into our findings, which will be investigated in a future study.

To conclude, this study provides evidence that the accuracy of eye movement in locating an item remembered in VSTM is significantly reduced in adults indicated to have MCI compared to normally aging older adults. Our data also indicate that eye movement deficit in locating an item remembered in VSTM in MCI individuals may appear earlier than VSTM deficits for object‐location binding, implying that eye‐tracking could serve as a valuable behavioral marker for detecting early cognitive decline. Future research could examine whether impaired saccadic accuracy arises from deficits in memory binding itself, failure in motor planning, or both. Longitudinal studies could determine whether these eye movement measures predict progression of MCI to dementia, offering a non‐invasive, behavioral marker for its early detection.

## Author Contributions

**Raju Sapkota**: conceptualization, methodology, formal analysis, investigation, resources, data curation, writing – original draft, writing – review and editing, supervision, project administration. **Monika McAtarsney‐Kovacs**: conceptualization, methodology, software, validation, formal analysis, investigation, resources, data curation, writing – review and editing, writing – original draft. **Ian van der Linde**: conceptualization, methodology, software, validation, investigation, resources, writing – review and editing, supervision, project administration, data Curation. **Shahina Pardhan**: conceptualization, methodology, investigation, resources, writing – review and editing, supervision, project administration, funding acquisition

## Funding

The authors have nothing to report.

## Ethics Statement

Ethical approval was obtained from Anglia Ruskin University (approval code: CIS201221).

## Consent

Informed consent was obtained from each participant prior to enrollment.

## Conflicts of Interest

The authors declare no conflicts of interest.

## Peer Review

The peer review history for this article is available at https://doi.org/10.1002/brb3.71021

## Institutional Review Board Statement

The study was conducted according to the guidelines of the Declaration of Helsinki and approved by the Ethics Committee of Anglia Ruskin University (ethics approval code CIS201221, date of approval January 4, 2022).

## Data Availability

Data may be obtained upon reasonable request to the corresponding author.
